# Recent Advances in Vertical Alveolar Bone Augmentation Using Additive Manufacturing Technologies

**DOI:** 10.3389/fbioe.2021.798393

**Published:** 2022-02-07

**Authors:** Cedryck Vaquette, Joshua Mitchell, Sašo Ivanovski

**Affiliations:** School of Dentistry, Centre for Orofacial Regeneration, Reconstruction and Rehabilitation (COR3), The University of Queensland, Herston, QLD, Australia

**Keywords:** extraskeletal bone, 3D-printing, BMP-2, bioceramic, polycaprolactone, bone regeneration

## Abstract

Vertical bone augmentation is aimed at regenerating bone extraskeletally (outside the skeletal envelope) in order to increase bone height. It is generally required in the case of moderate to severe atrophy of bone in the oral cavity due to tooth loss, trauma, or surgical resection. Currently utilized surgical techniques, such as autologous bone blocks, distraction osteogenesis, and Guided Bone Regeneration (GBR), have various limitations, including morbidity, compromised dimensional stability due to suboptimal resorption rates, poor structural integrity, challenging handling properties, and/or high failure rates. Additive manufacturing (3D printing) facilitates the creation of highly porous, interconnected 3-dimensional scaffolds that promote vascularization and subsequent osteogenesis, while providing excellent handling and space maintaining properties. This review describes and critically assesses the recent progress in additive manufacturing technologies for scaffold, membrane or mesh fabrication directed at vertical bone augmentation and Guided Bone Regeneration and their *in vivo* application.

## Introduction

Bone resorption is a phenomenon characterized by the volumetric reduction in viable bone tissue. Whilst osteoclast-mediated bone remodeling is an essential part of healthy bone metabolism, irreversible bone resorption can occur due to trauma or pathology within bone tissues (Teitelbaum, 2000). This is particularly problematic within the maxillofacial region, where surgical interventions, such as tooth extraction, can result in irreversible bone resorption leading to significant loss of bone volume. Consequently, a regenerative procedure for re-establishing the lost tissue and enabling the placement of prosthetic devices, such as dental implants, is required. Vertical bone augmentation aims to restore the previous levels of bone height, and is one of the most challenging surgical procedures in dentistry as it requires the formation and maintenance of extraskeletal bone (i.e., outside the newly established skeletal envelope) (Esposito et al., 2009; Urban et al., 2019).

Several existing techniques aimed at vertical bone augmentation, such as autologous block grafts, distraction osteogenesis, and guided bone regeneration combined with particulate grafts (GBR), have shown varying levels of success and are generally considered to be technique sensitive and clinically unpredictable (Urban et al., 2019). Indeed, whilst some commendable advances have been made in vertical bone augmentation, issues surrounding space maintenance, graft fixation, predictability of bone formation, and resorption still persist (Asa’ad et al., 2016). Other approaches are required to address these issues, and additive manufacturing (also known as three-dimensional (3D) printing) technology has been recently shown to have considerable potential to advance the field of vertical bone augmentation (Moussa et al., 2015; Carrel et al., 2016a; Carrel et al., 2016b; Sudheesh Kumar et al., 2018; Vaquette et al., 2021). Additive manufacturing enables the fabrication of porous biomaterials with an interconnected pore network in a layer-by-layer fashion and is additionally capable of fabricating customised patient-matched constructs. Advancements in bioceramic and polymer additive manufacturing techniques have paved the way for exploration into novel techniques in vertical bone tissue regeneration (Moussa et al., 2015; Carrel et al., 2016a; Carrel et al., 2016b; Ngo et al., 2018; Sudheesh Kumar et al., 2018; Vaquette et al., 2021).

*In vivo* application of such scaffolds has yielded some success in pre-clinical trials and generated strong interest within the field (Melchels et al., 2012; Khojasteh et al., 2013; Asa’ad et al., 2016). This review will briefly describe the various methods of 3D-printing for the manufacturing of 3D scaffolds (bioceramic and/or polymer), membranes, and patient matched metal meshes and then critically analyze the most recent studies utilizing additive manufacturing technologies for the purpose of alveolar vertical bone augmentation.

## Additive Manufacturing Technologies and Bone Regeneration

Significant progress in additive manufacturing technology (3D printing) has been achieved over the past twenty years. Multiple techniques ranging from powder ceramic to polymer fabrication have been developed and optimized, bringing the field to a level of technological competency whereby a diverse range of geometries can be fabricated relatively quickly, and with a high degree of dimensional accuracy and patient customization (Lipson et al., 2004; Feilden et al., 2016). Utilizing this technology in conjunction with conventional imaging techniques, such as computer tomography (CT) scanning, enables the manufacturing of scaffolds with identical geometric features to the host tissue (Melchels et al., 2011).

Synthetic scaffolds can be fabricated by a broad range of techniques, and the following Table 1 summarizes the main additive manufacturing technologies utilized for scaffold fabrication applied in bone regeneration.

For vertical bone augmentation, scaffolds must fulfill several essential criteria. The scaffold must be biocompatible and should not induce cytotoxicity, acute inflammation or any form of rejection or fibrous encapsulation (Langer and Vacanti, 1993). The material must be capable of integrating with the native tissue by facilitating infiltration of cells, i.e., progenitor cells or osteoblasts (Asa’ad et al., 2016). The scaffold should be highly porous with an interconnected porosity to facilitate rapid vascularization to support bone formation (Cruess and Cruess, 1982). Previous research demonstrated that a porosity ranging from 60 to 90% is appropriate for bone regeneration and that a pore size above 100 microns is required for enabling cell, tissue infiltration and vascularization (Karageorgiou and Kaplan, 2005). Interestingly, the pore size also seems to impact bone regeneration depending on the application, as a recent study reported (Ghayor et al., 2021). Indeed, it was demonstrated that a larger pore size was beneficial for vertical augmentation whereas a smaller pore size enhanced bone regeneration in a bony defect. Regardless of the internal architecture, the scaffolds must be self-supporting and mechanically robust for achieving appropriate space maintenance for bone ingrowth and to ensure it does not collapse upon mastication which force ranges from 50 to 200 N depending on age and position in the jaw (Edmonds and Glowacka, 2020). The current literature reporting on additively manufactured scaffolds for vertical bone augmentation can be divided into three major biomaterials groups: 1) bio-ceramics, 2) polymers, and 3) metals.

## Additively Manufactured Bio-Ceramic Constructs for Vertical Bone Regeneration

As mentioned, whilst bone grafting techniques result in desirable clinical success rates, the method is still prone to post-surgical complications, and handling can be difficult for large bone deficiencies (Nyström et al., 2002; Nyström et al., 2004). This has seen an evolution of bone grafting research into synthetic alternatives to better address resorption issues encountered with autogenous bone (Tamimi et al., 2006). Bio-ceramics are one such material group of interest due to their biocompatibility and efficacy to conduct and/or induce bone formation. Common materials of interest within this class are hydroxyapatite (HA), alpha tri-calcium phosphate (α-TCP), beta tri-calcium phosphate (β-TCP), and biphasic tricalcium phosphate (Asa’ad et al., 2016). Bio-ceramics are generally manufactured from a colloidal suspension which enables the shaping of an implant, and this part is called the “green body”. This “green body” is then subjected to high temperatures (typically 50–90% of the melting temperature) which gives the implant its final microstructure and properties (Lakhdar et al., 2021). This later step induces volumetric changes and therefore the final implant is smaller than the green body, which represents a significant challenge for the production of customized implants.

**TABLE 1:** Description of the various 3D-printing technologies.

Class	Manufacturing method	Operation	Compatible printing materials
Extrusion	Fused deposition modelling (FDM)	Material is heated until molten and is extruded through a printing head using either pressurized extrusion and screw based extrusion or a combination of both. The extruded material solidifies on contact with the base plate or previous layer, forming a filament commonly called strut.	Thermoplastic polymers, rubber, eutectic metals, clay (modelling and metal) Chua et al. (2010).
	Direct ink writing/robocasting (DIW)	A polymeric ink or a binder is extruded to manufacture scaffolds with a high resolution. Objects manufactured are initially soft and fragile thus accompanying support materials are often printed simultaneously. Drying, de-binding and sintering are required post printing for optimized mechanical characteristics Feilden et al. (2017).	Ceramics, ceramic and metal matrix composites, sol-gel, polymers Xu et al. (2006).
	Continuous filament fabrication (CFF)	Identical fabrication method to FDM but uses a polymeric filament which is locally molten in the printing head. The extrusion is generated by the utilization of rollers on the filament, thereby applying extrusion forces Tekinalp et al. (2014).	Polymer and Carbon based composites, nylon and Kevlar Tekinalp et al. (2014).
Polymerization by light	Continuous liquid interface production (CLIP)	Comprised of a bath with a transparent windowpane containing a photopolymer resin. An ultraviolet beam of light cures the resin layer-by-layer as the object is extruded vertically at a constant slow velocity. A nonpermeable oxygen membrane between the windowpane and resin bath allows the laser process to be continuous Tumbleston et al. (2015).	Photopolymer Tumbleston et al. (2015).
	Stereolithography (SLA)	Comprised of a bath with a transparent windowpane containing a photopolymer resin. An ultraviolet beam of light cures the resin layer-by-layer as the object is extruded vertically at a constant slow velocity. After each layer is cured, a blade component filled with resin is swept across the windowpane, providing new resin required to cure the next layer of printing Lipson et al. (2004).	Photopolymer Lipson et al. (2004).
Powder bed	Powder bed and inkjet head 3D printing (3DP)	An inkjet head deposits a liquid fusing substance which binds particles within the powder bed. Once a single layer has been completed, a new layer of powder is added on top of the completed layer and the process is then repeated iteratively layer-by-layer until the component is completed Shirazi et al. (2015).	Plaster, metallic alloy and ceramic powders Shirazi et al. (2015).
	Electron beam additive manufacturing (EBM)	An electron beam melts metal particles together within a bed of metallic powder inside a vacuumed environment. Once a single layer has been completed, a new layer of powder is added and the process repeated iteratively until a fully dense metallic object is formed Murr et al. (2012).	Metallic alloy powders Murr et al. (2012).
	Selective laser sintering (SLS)	A high-powered pulsating carbon dioxide laser fires onto a bed of powdered material which is preheated to slightly below melting point, subsequently binding the particles together. Like other powder bed technologies, SLS requires a fresh layer of powdered material to cover the completed cross-section iteratively until the 3D object is formed Williams et al. (2005).	Metal and ceramic powders, thermoplastic B. Williams et al. (2005).

Bio-ceramics characteristically exhibit excellent osteoconductive and sometimes osteoinductive properties (Barradas et al., 2011). This is largely due to the fact that they can be fabricated with coarse topography and surfaces suitable for the release of calcium and phosphate ions known for promoting the osteo-differentiation of progenitor cells. These materials are mostly utilized in a particulate form in combination with an occlusive membrane for bone regeneration in the oral cavity. While osteoconductive, the utilization of their granular form in the clinic is a significant challenge for handling and achieving adequate stability for vertical augmentation in the case of large bone deficiencies. In addition, the packing of the granular materials results in the formation of a highly tortuous porous network, which may impede rapid vascularization of large defects and delay bone formation as previously reported (Carrel et al., 2016b). Bio-ceramic 3D-printed scaffolds could potentially circumvent these issues as demonstrated in a clinical case which resulted in excellent structural maintenance and high bone formation seven years post-implantation (Mangano et al., 2021).

### Bio-Ceramic Scaffolds Manufactured by 3D Powder Printing

Several research endeavors have advocated a shift away from particulate grafting to programmable 3D fabricated bio-ceramics blocks (Gbureck et al., 2007; Klammert et al., 2010). Gbureck et al. developed a 3D powder printing technique utilizing a mixture of α/β Tricalcium Phosphate (TCP) particles which were reacted and bound together by spraying a phosphoric acid solution (Gbureck et al., 2007). This enabled a curing reaction of the TCP at room temperature, resulting in the creation of biodegradable secondary calcium phosphate matrices, namely brushite and monetite [dicalcium phosphate dihydrate (Brushite), and dicalcium phosphate anhydrate monetite (Monetite)]. The final phase composition of the fabricated material was a brushite phase (67%wt) and the remaining bioceramic was the unreacted α/β-TCP phase and a small amount of monetite. Further processing via hydrothermal reaction converted brushite components to monetite. The printed brushite scaffold and the thermally converted monetite were then compared *in-vivo* in an intramuscular rodent model. Surprisingly, the monetite scaffold underwent a more rapid degradation than the brushite. While brushite is a highly soluble phase and hence should have degraded first, a phase transition towards hydroxyapatite occurred via a dissolution/precipitation mechanism, thus rendering a proportion of the bio-ceramic block insoluble.

**FIGURE 1:**
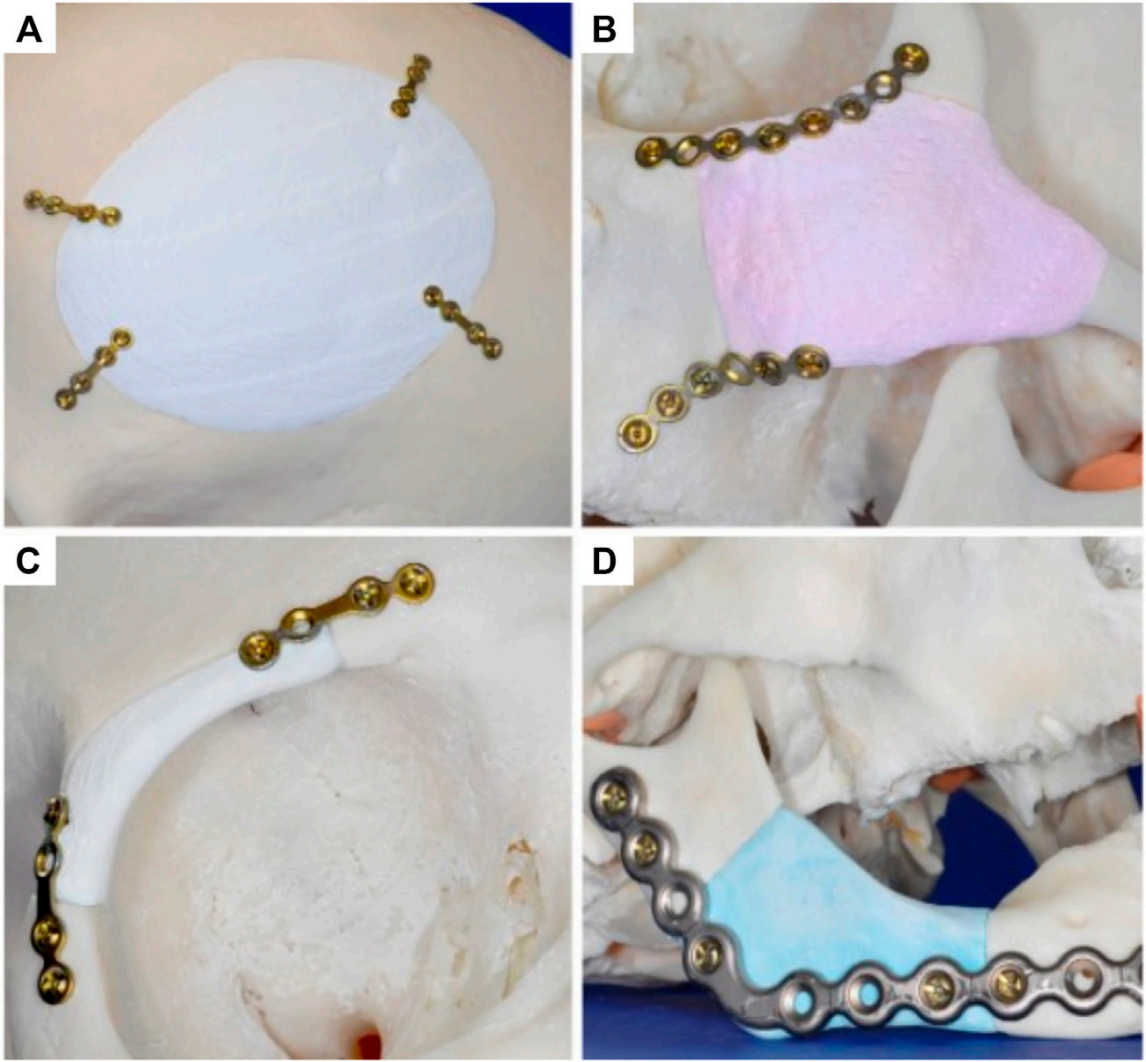
Manufacturing of anatomically accurate 3D-printed bioceramic scaffolds for bone regeneration demonstrating the versatility of the fabrication method for various applications in the craniofacial area. Reproduced with permission from (Klammert et al., 2010) **(A–D)**.

The versatility of this 3D-powder printing method was further demonstrated by manufacturing anatomically accurate scaffolds for potential craniofacial implantation (Figure 1) (Klammert et al., 2010). The study utilized a human cadaveric model featuring several defects that were imaged and numerically captured via computer tomography (CT), and further processed using computer aided design software (CAD) for STL file production and 3D-printing. Utilizing the 3D-powder printing technique previously described (Gbureck et al., 2007), 3D matrices of brushite (further hydrothermally converted to monetite) were fabricated matching the geometries of the defects. Although no quantitative data was provided, the study reported sound contour cohesion between the implant and defect, with some small overlapping areas which were later resolved by manual smoothing. Overall, this study demonstrated the ability of 3D-powder printed bio-ceramics to be accurately manufactured for a specific defect, which is a challenge for 3D printed bioceramics due to the significant dimensional changes occurring during the essential sintering process.

**FIGURE 2:**
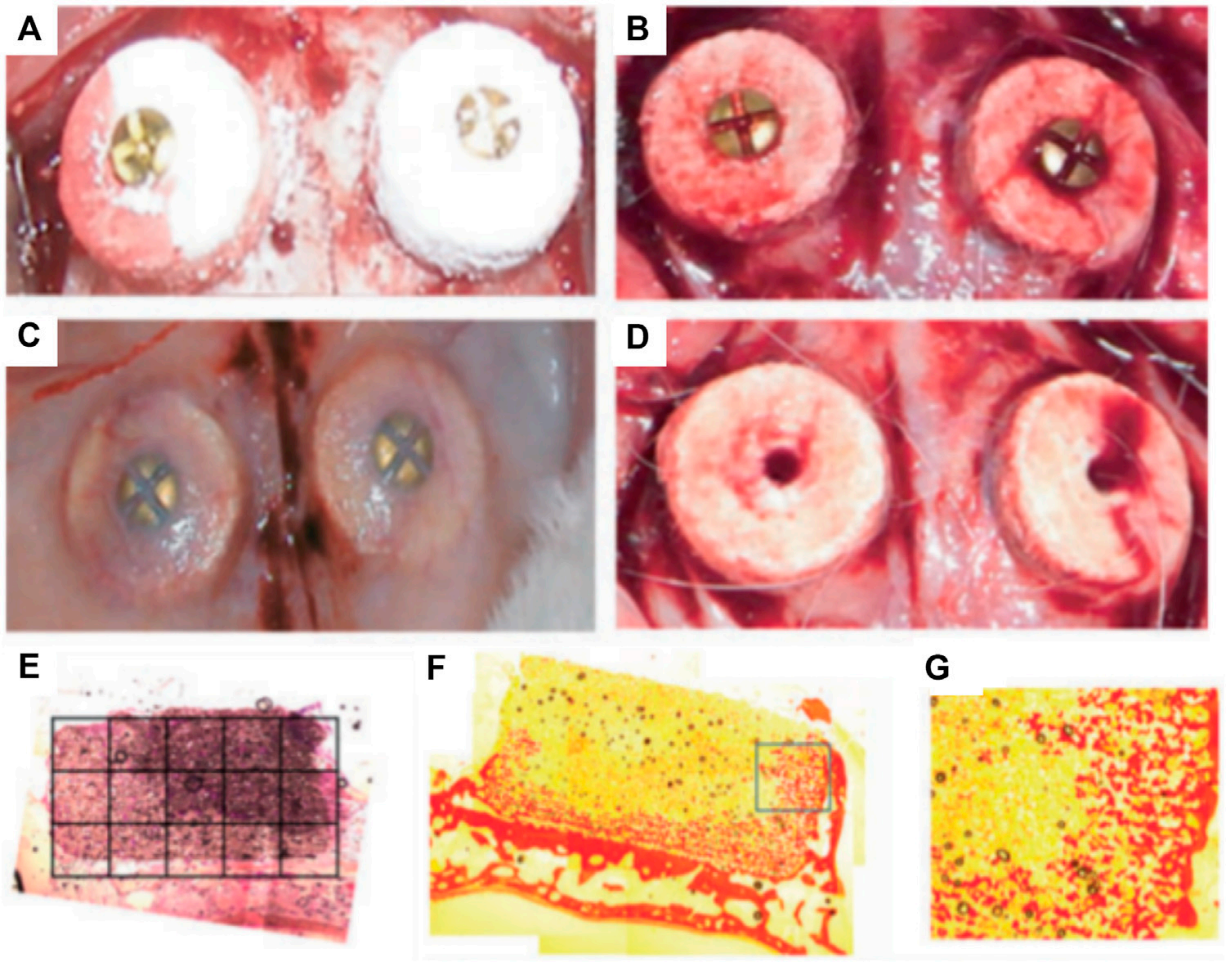
Influence of overall height of 3D-powder printing monetite scaffold in a lapine extraskeletal model. **(A)**: 3 and 4 mm high 3D-printed scaffolds were implanted and fixed using a fixation screw, **(B)**: shows the blood clot stabilization within the scaffold immediately after implantation. **(C)**: Specimen morphology 8 weeks post-implantation. **(D)**: Removal of the fixation screws at 8 weeks post-implantation, **(E–G)**: Tissue morphology as assessed by histology (picro-sirius staining) indicating a heterogenous distribution of the newly formed bone preferentially located near the resident bone and at the periphery of the scaffold. Reproduced with permission from (Torres et al., 2011).

The ability of the bioceramic scaffold to support vertical bone formation was further investigated in a lapine extraskeletal model (Tamimi et al., 2009). This study additionally evaluated the feasibility of fixing the 3D printed monetite block with craniofacial screws in an *in vivo* setting. The 3D-printed bio-ceramic scaffolds were trialed as an onlay graft and a 9 mm diameter and 2 mm thick monetite disc was compared to the performance of an autologous bone block of similar dimensions. Both structures were secured using a titanium osteosynthesis self-drilling screw placed at the center of the block. The samples were retrieved 8 weeks post-implantation and displayed no obvious signs of inflammation and were well integrated with the calvarium. However, the bone block demonstrated some resorption and histology analysis revealed intense osteoclastic activity at both the outer and inner regions of the autologous bone graft. The monetite scaffolds performed moderately well, resulting in bone formation preferentially on the lateral portions and in the area of the 3D-printed scaffold in direct contact with the calvarial bone. The bio-ceramic also displayed signs of extensive degradation and there was no significant difference in bone height when compared to the autologous bone block (Tamimi et al., 2009). A subsequent study was performed by Torres et al. using a similar 3D printed the monetite monolithic scaffold (Figures 2A–D) with a disc-shaped geometry, for assessing the influence of the bio-ceramic height upon vertical bone formation (3 and 4 mm height) (Torres et al., 2011). Here again, the blocks were fixated by an ostesosynthesis screw placed in a centrally located cylindrical hole and a period of 8 weeks was allowed for healing in an extraskeletal lapine model. Integration with calvarial tissue was deemed successful and histological analysis revealed that newly formed bone occupied around 40% of the blocks irrespective of their initial heights (Figures 2E–G) (Torres et al., 2011). Similar to the previous studies (Tamimi et al., 2009; Tamimi et al., 2014), the majority of bone was located in the proximity of the resident calvarial bone and at the periphery of the 3D-printed scaffold. This heterogeneous bone formation was attributed to the poorly interconnected porous network throughout the scaffold, which subsequently impeded vascularization and hence bone formation.

Overall, these studies highlighted the limitations of 3D-powder printing of monetite scaffolds, which despite appropriate fixation being achieved, could not support extensive bone formation, most likely due to the relatively low porosity and lack of pore interconnection preventing vascularization.

**FIGURE 3:**
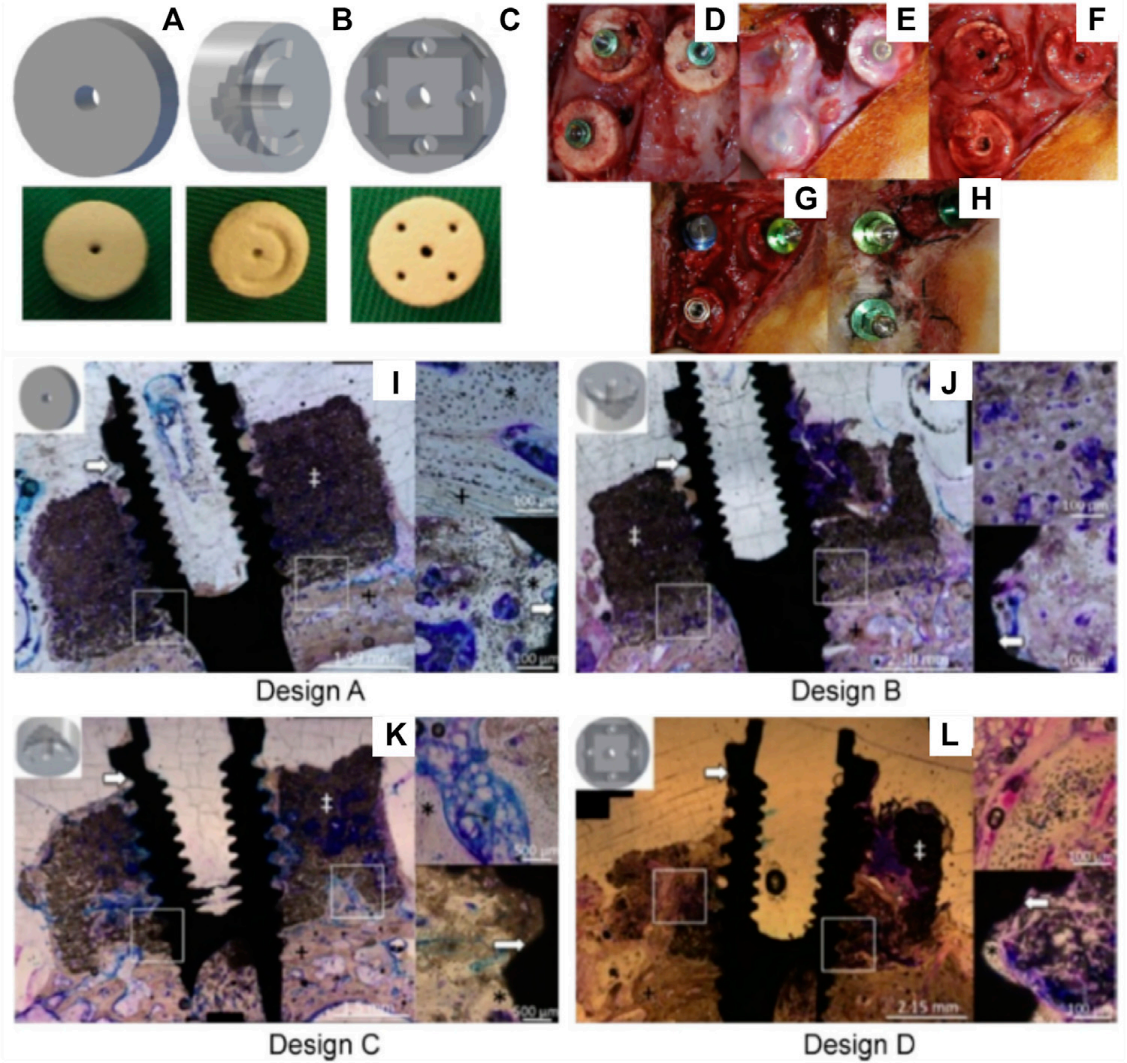
Assessment of the bone formation performance of 3D-powder printing including various microporosity in the forms of semi-circular grooves and channels in a lapine extraskeletal model. **(A)**: Design A “control” 3D-printed monetite scaffold, **(B)**: Design B and C including a semi-circle shaped groove spanning half the diameter of the scaffold, with the semi-circle in configuration B facing away from the calvarial bone, whilst the semi-circle in configuration C faced the calvarial bone, **(C)**: Design D consisting of an array of eight interconnected channels creating multiple apertures on each surface of the scaffold, **(D)**: surgical fixation of the scaffold, **(E)**: surgical re-entry at 4 weeks post-implantation, **(F)**: removal of the fixation screws, **(G)**: Dental implant placement, **(H)**: appearance of the specimens at 4 weeks post-implant placement. **(I–L)** Tissue morphology as demonstrated by histology, indicating that the presence of the channels was beneficial to bone formation, although the overall amount of bone tissue was not greatly increased and its distribution remained heterogeneous. Reproduced with permission from (Tamimi et al., 2014).

Consequently, a scaffold with increased interconnectivity by including channels within its core was developed (Tamimi et al., 2014). Several configurations were assessed as shown in Figures 3A–C. Design A consisted of an unmodified monetite 3D-printed scaffold. Designs B and C included a semi-circle shaped groove spanning half the diameter of the scaffold, with the semi-circle in configuration B facing away from the calvarial bone, whilst the semi-circle in configuration C faced the calvarial bone. Design D consisted of an array of eight interconnected channels creating multiple apertures on each surface of the scaffold. The scaffolds were implanted in a lapine model, a surgical re-entry was performed 4 weeks post-implantation for enabling the placement of titanium dental implants (Figures 3D–H), and osseo-integration was allowed for a further 4 weeks. At completion of the study (8 weeks total) it was demonstrated that some bone had formed preferentially in the proximity of the host bone and in the vicinity of the channels (Figures 3I–L). However, configurations C and D that had channels in direct contact with the host bone displayed the largest amount of bone formation. Configuration A and B resulted in the lowest bone formation, demonstrating that the presence of the macroscopic channels in the other designs improved vascularization and hence bone formation ability of the scaffolds. Histology confirmed that the dental implant was osseo-integrated with any newly formed vertical bone that was present. The study concluded that modifying the geometry of the scaffolds enhanced uniform bone regeneration. However, the most medial and superior sections of all scaffold configurations exhibited little or no bone formation (Tamimi et al., 2014). While the introduction of interconnected macro-channels was beneficial for bone formation, the later was mostly restricted to portions of the scaffold where a rapid vascularization occurred (such as in the channels or in the peripheral aspects of the scaffold). Despite the high bioactivity of bio-ceramic materials, the lack of significant bone in growth in the powder 3D printed scaffolds, featuring a low porosity, small pore size and a tortuous porous network, exemplifies the importance of the construct internal porous architecture in facilitating vascularization, and subsequent bone formation. Scaffold fabrication via a direct printing approach can circumvent these issues and produce a construct with a porous network that is more favorable for uniform bone regeneration.

### Bio-Ceramic Scaffolds Manufactured by Extrusion 3D Printing

**FIGURE 4:**
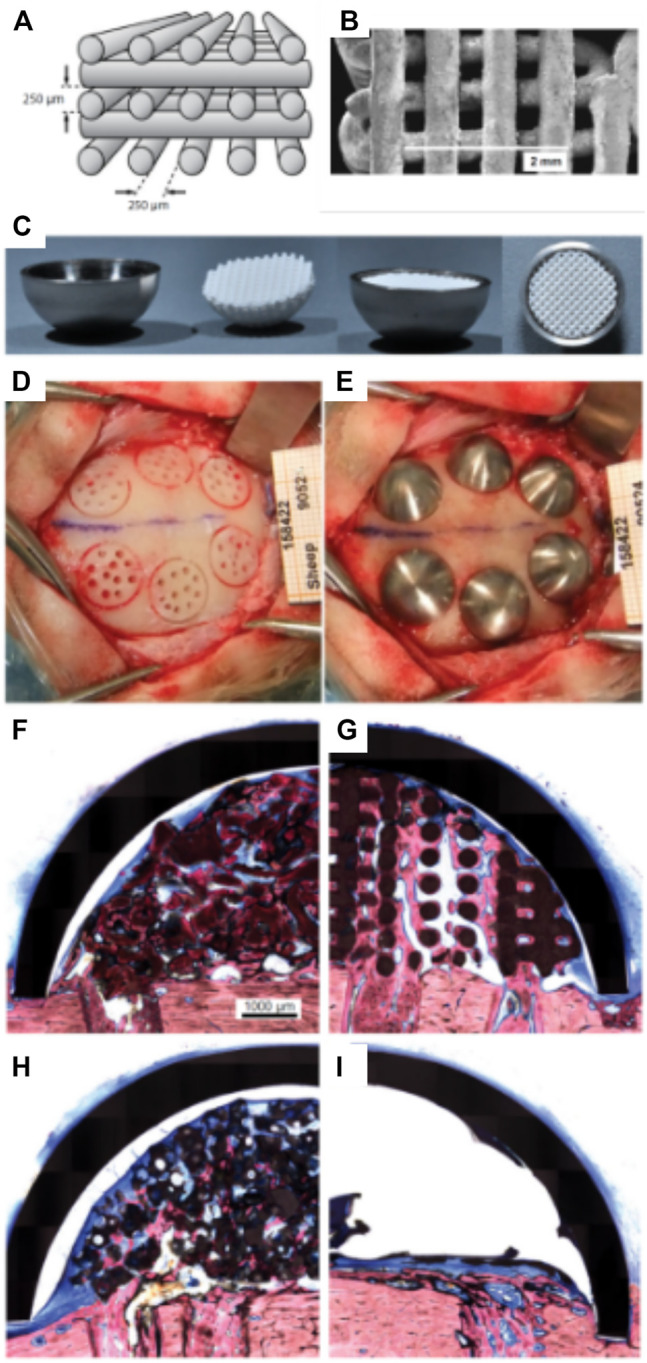
Bio-ceramic scaffold manufacturing via direct extrusion printing. **(A)**: Schematic representation of the 3D-printed Osteoflux bio-ceramic, **(B)**: Morphology of the 3D-printed scaffold as imaged using Scanning Electron Microscopy. **(C)**: implantation system featuring a titanium dome acting as an occlusive barrier. **(D)**: Preparation of the implantation site using a sheep extraskeletal model. **(E)**: pictures of the implanted domes containing the various groups. **(F–I)** histology of the specimen at 8 weeks post-implantation demonstrating the excellent bone forming ability of the 3D-printed scaffold **(F)**: Bio-Oss, **(G)**: 3D-printed Osteoflux, **(H)**: Ceros, **(I)**: empty dome (blood clot). Reproduced with permission from (Carrel et al., 2016b).

Carrel et al. confirmed that a highly porous structure, manufactured by extrusion 3D-priting and hence possessing a fully interconnected macropore network (Carrel et al., 2016b), performed better than other randomly organized geometries with lower porosities (Tamimi et al., 2006; Tamimi et al., 2009; Torres et al., 2011; Tamimi et al., 2014). This comparison was undertaken by assessing the performance of three different biomaterials: a 3D-printed bio-ceramic Osteoflux (OF), and two commercially available particulate bone grafting materials, Bio-Oss (BO) and Ceros (CO), with particle size of 0.25—1 mm, and 0.5–0.7 mm, respectively. The OF scaffolds were fabricated via 3D-printing using a calcium phosphate mixture composed of a calcium deficient hydroxyapatite and α-TCP, enabling the manufacturing of 400 µm diameter filaments regularly ordered to form 250 µm pores (Figures 4A,B). The scaffold or the granulate materials were housed under a titanium dome (Figure 4C) and subsequently implanted extraskeletally on the skull of sheep (Figure 4D).

**FIGURE 5:**
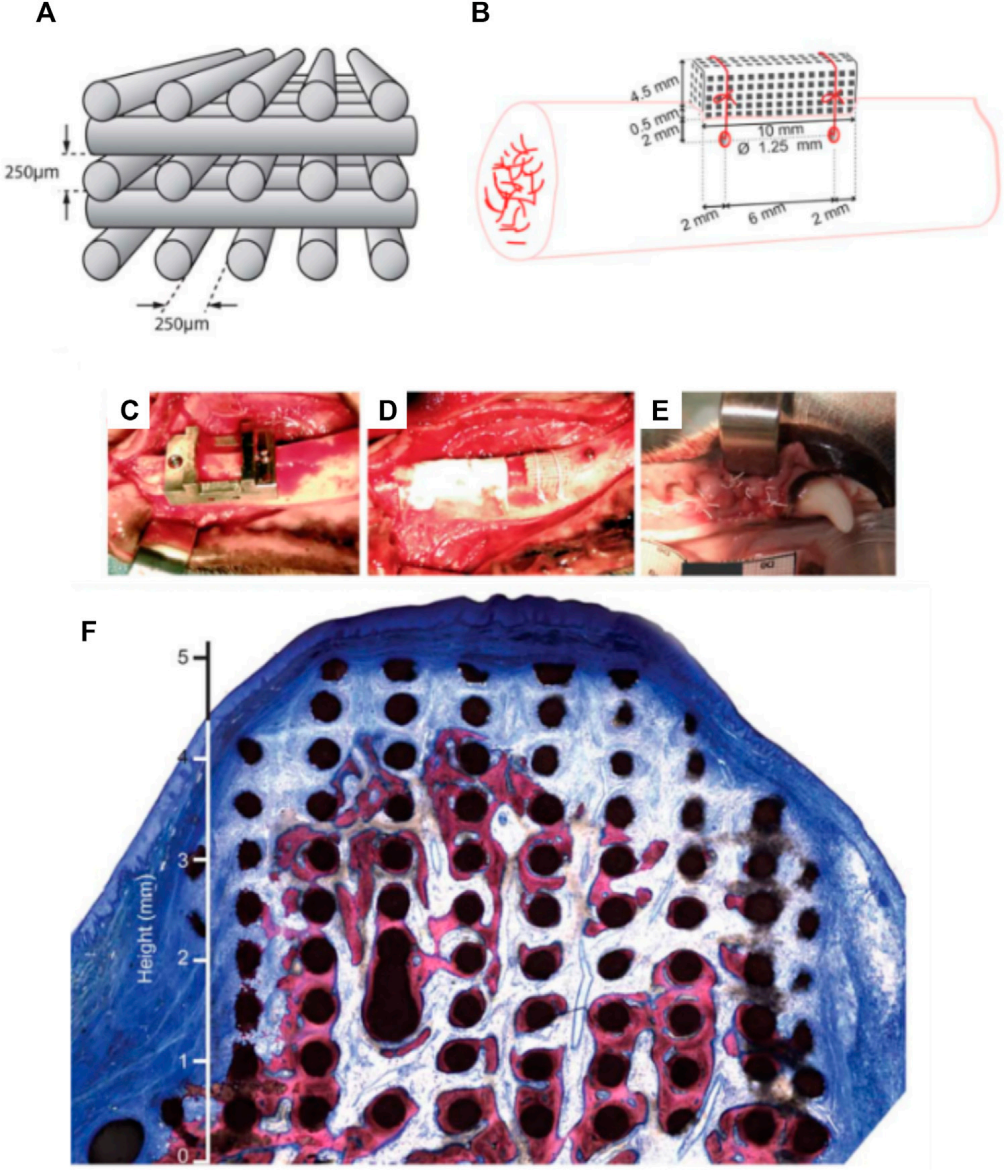
3D-printed bio-ceramic scaffold manufactured by a direct printing method. **(A,B)**: Schematic description of the scaffold porosity and dimensions, as well as the implantation in a canine model. **(C–E)**: Implantation of the 3D-printed scaffold and coverage by a collagen membrane. **(F)**: histology of the vertically augmented bone 8 weeks post-implantation. Reproduced with permission from (Carrel et al., 2016a).

The specimens were retrieved 8 weeks post-implantation and histomorphometry revealed that the new bone area in the OF (Figure 4G) samples was approximately 20% of the total dome area, BO (Figure 4F), and CO (Figure 4) displayed around 14% bone fill, while the empty dome resulted in negligible bone formation (Figure 4I). At 16 weeks post-implantation, all groups (other than the Empty group) displayed similar bone fill at around 40%, indicating that the 3D-printed scaffold enabled earlier bone formation. Interestingly, the 3D-printed scaffold also enabled increased bone height at early time points when compared to the particulate material. This was attributed to the interconnected highly porous lattice structure of the scaffold that permitted enhanced vascularization at the superior regions of the scaffold. This indicates that the 3D-printed scaffolds were architecturally designed to be conducive to both horizontal and vertical bone augmentation. In contrast, the randomly organized porous networks of particulate materials had an inferior capacity to support vascularization, resulting in delayed bone formation when compared to the 3D-printed scaffolds. These findings were confirmed in a subsequent study that assessed the performance of 3D-printed Osteoflux scaffolds in a more clinically relevant canine model (Carrel et al., 2016a). This model incorporated vertical bone augmentation in a surgically created edentulous area of a dog mandible. This was performed using a 3D-printed scaffold with dimensions of 10 mm length, 10 mm width, 5 mm height (Figures 5A,B). Four shallow bony defects were created via the removal of molars and premolars (specifically P1-4, M1, both left and right) (Figures 5C–E) and the 3D-printed scaffolds were implanted as an onlay graft. They were biomechanically secured using two Teflon loops inserted in two transcortical tunnels (1.25 mm in diameter), indicating perhaps that conventional fixation using titanium screws was not possible. A collagen membrane covered the scaffolds for a subsequent 8-weeks healing period, consistent with standard clinical practice.

While the study utilized only one animal, the proof of concept was nevertheless established. No sites showed evidence of inflammation and the scaffolds were well integrated with the native tissue (Figure 5F), and the newly formed bone was highly vascularized. filling approximately one third of the elevated volume and reaching heights between 4 and 5 mm. Specifically, when compared to other ceramic scaffolds in the field, both studies by Carrel et al. demonstrated a higher volume of bone that was also more uniformly distributed throughout the scaffold. This was attributed to the superior vascularization capacity of the scaffold, particularly in its superior segments, facilitated by the highly interconnected porous network (Carrel et al., 2016a; Carrel et al., 2016b). The main disadvantage of this bio-ceramic 3D-printed scaffold is likely to arise from its inability to be biomechanically secured using fixation screws, which would be problematic in the clinical setting (Carrel et al., 2016a; Carrel et al., 2016b).

### Bio-Ceramic Scaffolds Used as Delivery Vehicles for Osteogenic Molecules

**FIGURE 6:**
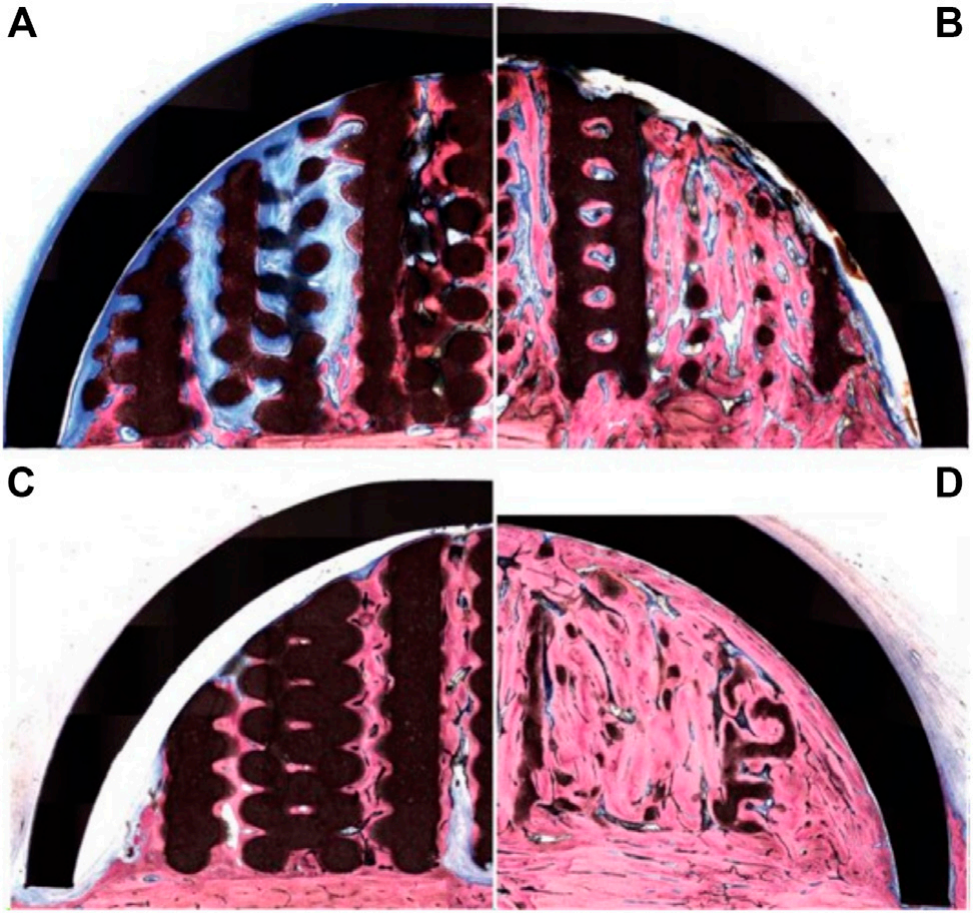
3D-printed bio-ceramic scaffold combined with Bone Morphogenetic Protein-2 for vertical bone formation. **(A)**: pristine 3D-printed bio-ceramic scaffold and **(B)**: BMP-2 loaded scaffold at 8 weeks post-implantation, **(C)**: pristine 3D-printed bio-ceramic scaffold and **(D)**: BMP-2 loaded scaffold at 16 weeks post-implantation demonstrating higher bone formation in the BMP-2 loaded scaffold along with advanced degradation of the bio-ceramic scaffold (in black) at the 16 weeks timepoint. Reproduced with permission from (Moussa et al., 2015).

Whilst bio-ceramic grafts possess excellent osteoconduction capacity, their bone formation ability can be further improved by combining them with biological cues. The incorporation of bone morphogenetic protein 2 (BMP-2) in the scaffold is a potent means of increasing osteogenesis due to its ability to trigger osteoblastic differentiation in a wide range of cell types (Meejung and Senyon, 2011). This strategy was explored by Moussa et al. (2015) using the 3D-printed bio-ceramic scaffold described in the previous section. The performance of the BMP-2 loaded scaffold was assessed using the same ovine extraskeletal bone regeneration model. To this end, the scaffold, loaded with 100 µg recombinant human BMP-2, was implanted for 8 and 16 weeks and compared to the unloaded scaffold filled with a natural blood clot. The BMP-2 primed scaffold resulted in higher bone formation at both 8- and 16-weeks post-implantation (Figures 6A–D), and degradation of the bio-ceramic scaffold was further accelerated by the presence of the growth factor (Figure 6D). Indeed, at the 16 weeks timepoint, only trace amounts of the scaffold material were observable. While the incorporation of BMP-2 results in excellent vertical bone formation, likely problems of achieving fixation of such a brittle scaffold, as highlighted in the previous section, may still hinder clinical translation.

## Additively Manufactured Polymeric Constructs for Vertical Bone Regeneration

The development of more flexible polymeric scaffolds has been advocated as an alternative for circumventing the brittleness of bioceramic scaffolds and their poor fixation ability. The following sections describe the most recent advances in the use of additively manufactured polymeric structures for vertical bone regeneration.

### Polymeric Porous 3D Printed Scaffolds for Bone Regeneration

Polymer printing is capable of building a refined interconnected porous network which facilitates neo-vascularization and a provide an environment suitable for osteogenesis to occur. In addition, the utilization of flexible and ductile polymers enables adaption of the defect shape and allows usage of titanium screws for biomechanical fixation. Several proof-of-concept clinical reports using 3D-printed scaffolds for oro-dental tissue regeneration in socket preservation (Goh et al., 2015) and periodontal regeneration (Rasperini et al., 2015) have been reported, however, this approach has not been translated yet to the clinic for vertical alveolar bone formation. Pre-clinical studies using various animal models that explore the potential of additively manufactured polymeric scaffolds for vertical alveolar bone augmentation are discussed below.

An early report for vertical bone augmentation was published in 2013 by Khojasteh et al. whereby a 3D-printed β-TCP/polycaprolactone (PCL) scaffold (20 × 10 × 10 mm^3^) was implanted in the mandible of dogs (Khojasteh et al., 2013). The scaffold was seeded with bone marrow mesenchymal stem cells 24 h prior to implantation and healing was allowed for 8 weeks. The cell-laden scaffold performed significantly better when compared to the scaffold without cells with 50 and 20% bone fill, respectively. The poor performance of the scaffold without a biological additive highlights the bioinert nature of the PCL, even when blended with inorganic fillers. Interestingly, this study also reported that the scaffold was fixed using a titanium screw, demonstrating the favorable properties of the polymeric material in terms of facilitating the clinical handling and utilization of additively manufactured constructs.

**FIGURE 7:**
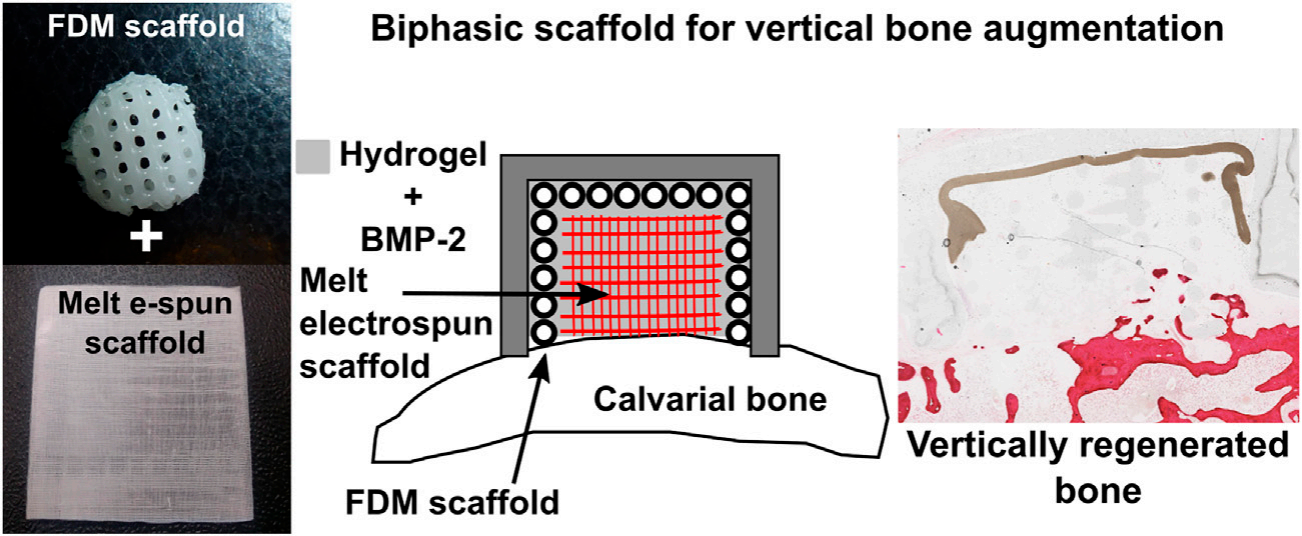
Additively manufactured biphasic scaffold for vertical bone augmentation using a biodegradable polymer, polycaprolactone loaded with a hydrogel. Reproduced with permission from (Sudheesh Kumar et al., 2018).

Kumar et al. further explored the feasibility of a scaffold comprised entirely of polycaprolactone (PCL) and functionalized with BMP-2 in a rabbit model (Sudheesh Kumar et al., 2018). This study utilized a biphasic scaffold consisting of a 3D-printed mechanically robust outer shell, mimicking the cortical plate, into which highly porous melt electrospun scaffolds, mimicking cancellous bone, were inserted. The rationale behind this concept was to promote rapid vascularization within the interior component of the scaffold, whilst the exterior component provided mechanical integrity necessary for space maintenance. The exterior shell component was fabricated through conventional fused deposition modelling (FDM) while the interior component was fabricated through melt electrowriting (Brown et al., 2011; Brown et al., 2012) (Figure 7), producing fibres of 400 and 10 µm in diameter, respectively. Additionally, an occlusive dome made of poly-L-lactic acid (PLLA) was utilized to prevent fibrous tissue infiltration, consistent with the principles of guided bone regeneration. The study also investigated the incorporation of a hydrogel loaded with recombinant BMP-2 within the scaffold, implanted in an extraskeletal lapine model. While a groove to accommodate the dome was prepared, no transcortical perforations were made, drastically limiting blood clot formation which delayed the initiation of the healing cascade, and therefore neovascularization and bone formation within the scaffold. Accordingly, whilst the scaffold exhibited biomechanical stability and space maintenance, it failed to result in significant new bone formation beyond the resident bony bed. Nonetheless, the concept showed sufficient potential to warrant further investigation for enhanced efficacy following optimization of both the scaffold and the *in-vivo* model.

**FIGURE 8:**
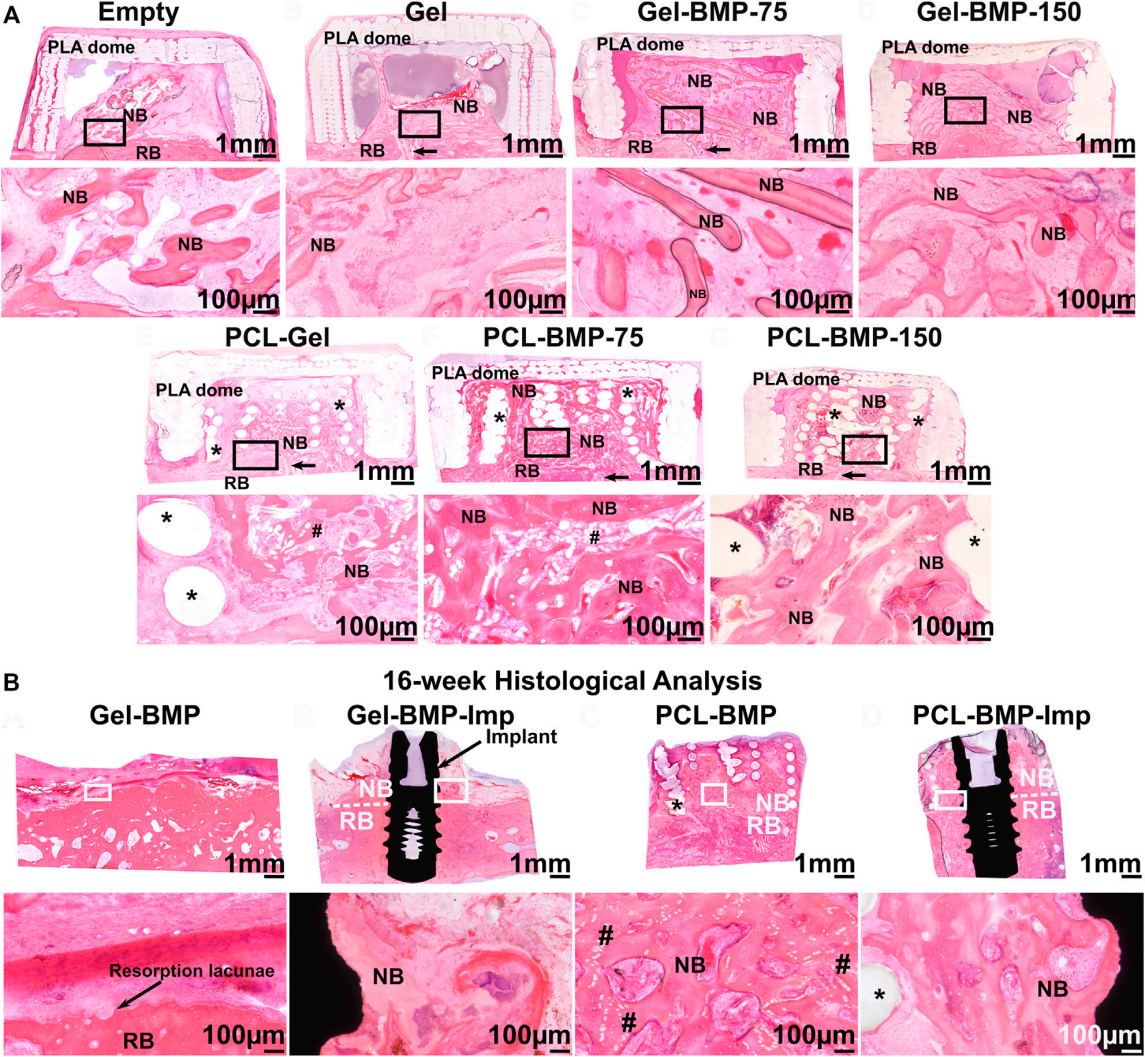
Efficacy of a biphasic scaffold fabricated by converging two additive manufacturing technologies (3D printing for the outer shell provides mechanical stability and melt electrospinning writing for the inner portion imparting high porosity, thereby facilitating vascularization, and bone formation) **(A)**: Extraskeletal bone formation using an ovine calvarium onlay graft model, demonstrating the necessity of BMP-2 to achieve significant bone formation and showing that the presence of the scaffold resulted in enhanced osteogenesis due to its excellent Gel/BMP-2 retention ability. **(B)**: Assessment of bone maintenance subsequent to surgical re-entry and implant placement in the previously vertically augmented bone. This revealed that the presence of the biphasic scaffold prevented early bone resorption which is a frequent event upon implant placement. Reproduced with permission from (Vaquette et al., 2021).

In a follow up study performed in an extraskeletal ovine calvarium model, the regenerative potential of the PCL 3D-printed/melt electrowritten biphasic scaffold was further explored for the formation and maintenance of vertically augmented bone (Vaquette et al., 2021). A 2-stage study first investigated the effect of the scaffold and BMP-2 dose on bone formation. Subsequently, bone maintenance and implant osseointegration were assessed, including surgical re-entry and placement of a dental implant. In the first step, seven configurations were examined: an empty dome, a biphasic scaffold functionalized with a gelatine hyaluronic hydrogel, a biphasic scaffold functionalized with a gelatine hyaluronic hydrogel containing 75 or 150 µg of BMP-2, the gelatine hyaluronic hydrogel alone or containing 75 or 150 µg of BMP-2. Study outcomes demonstrated that the presence of the scaffold improved vertical bone regeneration, potentially due to the hydrogel retention capacity of the scaffold, as shown in Figure 8A. Interestingly, the dose of BMP-2 did not make a significant difference in the volume of extraskeletally-formed bone, suggesting that there is a threshold in the dose of BMP-2 for initiating bone formation in a given defect volume, and that any addition of the growth factor may not result in enhanced bone formation. The second stage of the study involved placement of a dental implant in either bone previously formed in the BMP-2 containing hydrogel, or in the BMP-2 functionalized biphasic scaffold. Eight weeks of healing post-implantation was allowed, resulting in full resorption of the bone when the biphasic scaffold was absent (Figure 8B). This demonstrated with high reproducibility that the presence of a long-term space maintaining scaffold prevents early bone resorption and imparts enhanced dimensional stability to the elevated bone. A longer healing period will determine whether the elevated bone can be maintained over extended periods or after the PCL scaffold has fully degraded. This would require 3–5 years to complete degradation as PCL is a slowly degradable polymer (Lam et al., 2007; Bartnikowski et al., 2019).

Although not directly related to vertical bone augmentation, Goh et al. demonstrated in both an preclinical and a pilot clinical study that a 3D-printed PCL scaffold was capable of providing space maintenance in fresh extraction sockets (Goh et al., 2014; Goh et al., 2015). The study consisted of thirteen randomly selected patients, with seven patients acting as the control group with no space filler, whilst six patients received a PCL scaffold implanted into the tooth socket. The study assessed newly formed bone 6 months post-surgery via removal of a central segment of bone and subsequent micro-CT and histological analysis (prior to stage II surgery). Bone height, particularly at the mesio-buccal aspect, was superior in the patients that had received a polycaprolactone scaffold (Goh et al., 2015). Although both the control and PCL groups underwent bony ridge resorption, the PCL scaffold was able to limit this. This is likely attributed to the ability of the PCL scaffold to retain its geometry over the 6-month period post-surgery. Whilst this is favourable for initial space maintenance, the lack of material resorption may inhibit new bone growth and impede the long-term success of the procedure.

Other approaches have utilized additive manufacturing as a tool for fabricating guided bone regeneration membranes in conjunction with particulate materials for vertical bone augmentation. These are discussed further in the following sections.

### Guided Bone Regeneration Using Additively Manufactured Polymeric Membranes

In a series of publications Shim et al. investigated the effect of 3D-printed membranes with small pore size for GBR application (Kim et al., 2014; Shim et al., 2014; Shim et al., 2015; Shim et al., 2017). Initially, the membrane was composed of a slow degrading polymer (polycaprolactone, PCL) and a more rapidly degrading polymer [poly-lactic-co-glycolic acid, PLGA (85/15)] (Kim et al., 2014; Shim et al., 2014; Shim et al., 2015), whose half-life *in vivo* was around 9 weeks as previously reported for a porous sponge (Lu et al., 2000). Within the field of vertical bone augmentation, membranes can be utilized both as a “barrier” that mitigates against an infiltration of connective tissue and a supporting structure for space maintenance. Shim et al. explored hybrid polymer-ceramic membrane technology in conjunction with growth factors, thus developing a bioactive membrane targeting vertical bone augmentation in a lapine calvarial defect model. These 3D-printed membranes were manufactured by blending polycaprolactone with polylactic-co-glycolic acid and beta tricalcium phosphate (PCL/PLGA/β-TCP) and this polymer blend was subsequently 3D-printed (Shim et al., 2014). The pores of the 3D-printed membrane were filled with a collagen solution containing rhBMP-2. The membrane (10 mm diameter x 0.5 mm height) was then placed over an 8 mm surgically-created calvarial defect, and fixed using titanium screws. This study compared three groups: control, PCL/PLGA/β-TCP, and PCL/PLGA/β-TCP/rhBMP-2. Bone regeneration was assessed at four- and eight-weeks post-implantation, and while the control group did not exhibit any substantial bone formation, both membranes performed well, with significantly more bone formed with the BMP-2 loaded membranes (Shim et al., 2014). Interestingly, the 8-week rhBMP-2 specimens displayed almost full bone fill of the interstitial space demonstrating the effectiveness of integrating rhBMP-2 into polymeric membranes for vertical bone regeneration. Here again, the utilization of polymeric 3D printed composites may be a more advantageous choice for scaffold material due to their flexibility and enhanced handling ability compared to bio-ceramics.

**FIGURE 9:**
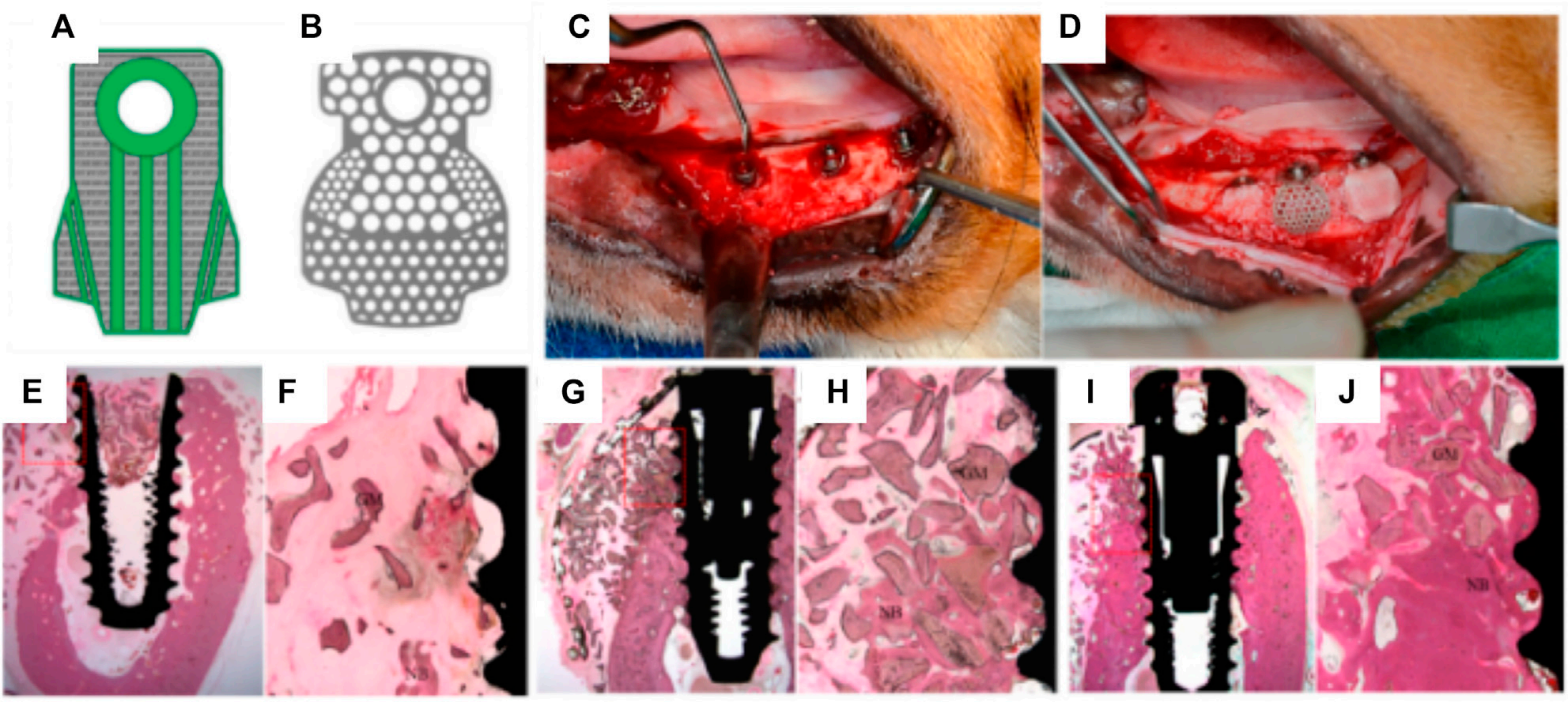
Efficacy of 3D-printed composite GBR membranes for bone formation 8 weeks post implantation. **(A)**: 3D CAD model design of the PCL/PLGA/β-TCP membrane; **(B)**, Design of pre-formed titanium mesh. **(C,D)**: Surgical protocol showing the placement of dental implant overlayed by the 3D-printed membrane. **(E–J)**: tissue morphology of the various groups **(E,F)**: no membrane, **(G,H)** 3D-printed membrane, **(I,J)**: Titanium membrane. Reproduced with permission from (Shim et al., 2015).

Shim et al. further compared the bone forming capacity of PCL/PLGA/β-TCP membranes with that of a titanium mesh, in a canine mandibular defect model (Shim et al., 2015). Due to its high biocompatibility, mechanical and space maintaining properties, titanium fulfills most of the design criteria of an ideal scaffold for guided bone regeneration, and as such, titanium reinforced meshes are currently used for vertical bone augmentation in the clinical setting. The *in vivo* study involved placement of a dental implant, over which a pre-formed 3D-printed scaffold or titanium membrane was positioned in order to provide space maintenance and contain the particulate bone grafting biomaterials utilized in this procedure. Following an 8-weeks healing period, the degree of bone formation and overall osseointegration was reported as being comparable between the two experimental groups (Figure 9).

However, signs of resorption were observed in the PCL/PLGA/β-TCP membrane group, likely attributed to the rapid degradation of the PLGA polymer which was the main polymer in the scaffold (the PCL:PLGA:β-TCP membrane ratio was 2:6:2). As anticipated, no material resorption was observed within the titanium mesh, and this is consistent with this structure requiring additional surgery for removal in the clinical setting. Whilst Shim et al. recorded no signs of inflammation at 8 weeks post-implantation (Shim et al., 2015), the rapid degradation of the PLGA leading to a potential burst release of acidic degradation by-products may trigger an unfavorable inflammatory reaction. In addition, PLGA degradation may also reduce the overall mechanical properties of the membrane and consequently undermine its mechanical and space maintenance properties in the longer term.

**FIGURE 10:**
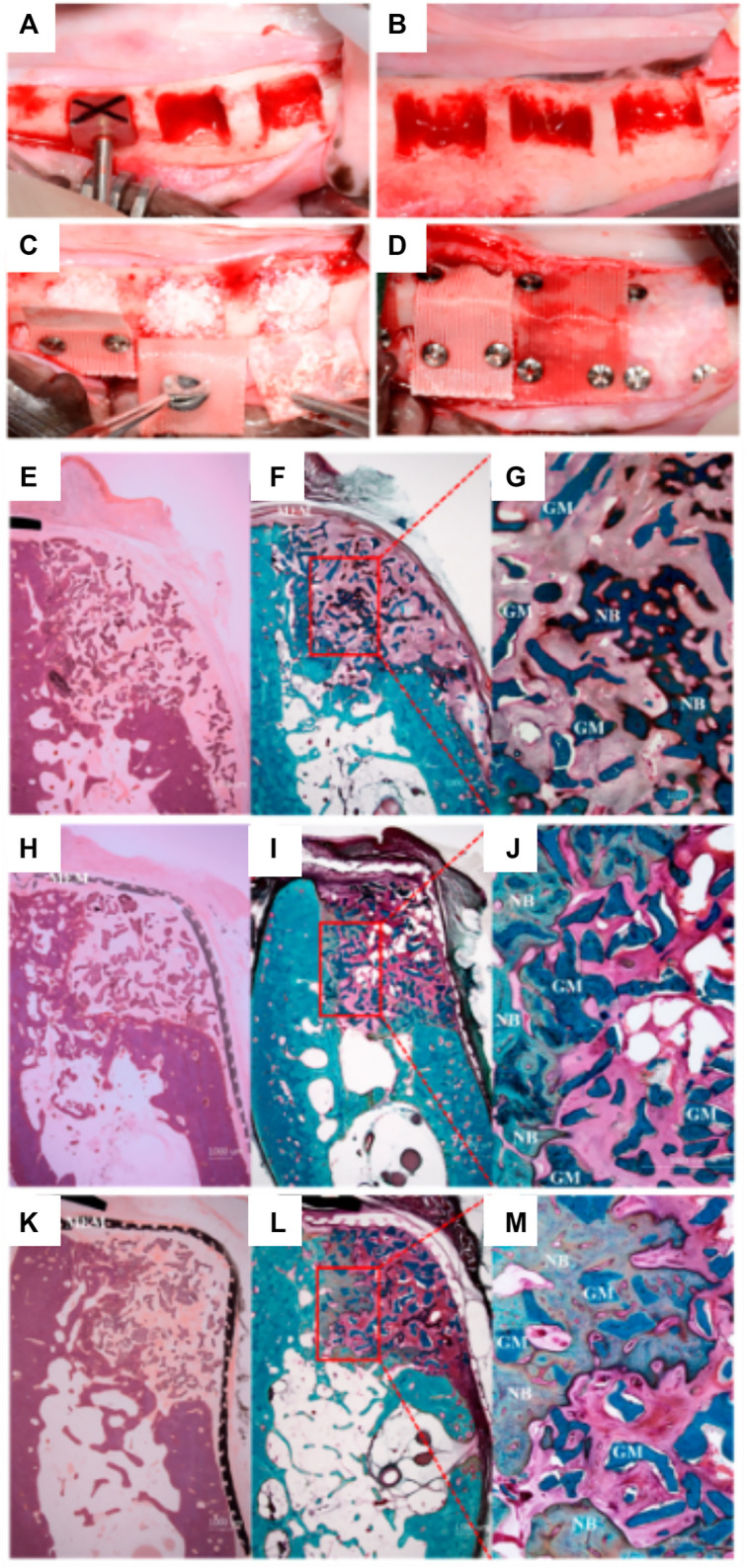
Comparison of PCL and PLC-BTCP 3D-printed membranes to a collagen membrane in a canine mandibular defect. **(A,B)**: Surgical preparation of the site in order to create standardized defects, (length: 7 mm, height: 5 mm, depth: 5 mm) **(C)**: Implantation of the bone grafting particulate materials, **(D)**: placement of the various membranes fixed using titanium screws. Tissue morphology as demonstrated using Hematoxylin and eosin **(E, H, K)** stain and Goldner Trichrome stain **(F,G,I,J,L,M)** for collagen **(E–G)**, PCL **(H–J)**, PCL/b-TCP membrane **(K–M)**. Reproduced with permission from (Shim et al., 2017).

Shim et al. therefore explored two further refined versions of the polymer membranes (Shim et al., 2017); PCL only and PCL/b-TCP. As with the previous studies, the primary aim was to assess the space-maintaining capacity and overall regenerative properties of the membrane. In line with their previous study, the PCL-only and PCL/b-TCP 3D-printed membranes were implanted over a mandibular defect in a canine model (Figures 10A–D) and compared to a collagen membrane. A standardized defect geometric volume of 175 mm^3^ was generated in six mandibular locations, in three different animals. The membranes were fastened by titanium pins, with bovine graft particulate placed underneath each membrane and healing occurred over 8 weeks.

Interestingly, and despite the difference in initial pore size between the collagen and the 3D-printed membranes, both performed similarly, and no significant differences were found between the respective amount of bone formed (Figures 10E–M). The main advantages of the PCL or composite 3D-printed membrane is the long-term space maintenance and the enhanced handling ability when compared to a hydrated collagen membrane that loses most of its stiffness and handling ability once in contact with biological fluids. Whilst this was a promising result and effectively demonstrated the efficacy of a partially occlusive polymer/ceramic composite scaffold, the method still relied upon bone particulate grafting for ensuring bone formation and is therefore still prone to handling and stability issues. In addition, the scaffold was utilized for covering a confined defect and therefore not assessed for vertical bone augmentation, which is far more demanding. Indeed, although the handling of these polymeric membranes is enhanced by their flexibility, inadequate distribution of stresses throughout the membrane is problematic and could potentially lead to failure. Further, the shape of the resulting elevated volume is poorly controlled and may not recapitulate the original anatomical features of the jawbone.

## Additively Manufactured Titanium Meshes for Vertical Bone Regeneration

**FIGURE 11:**
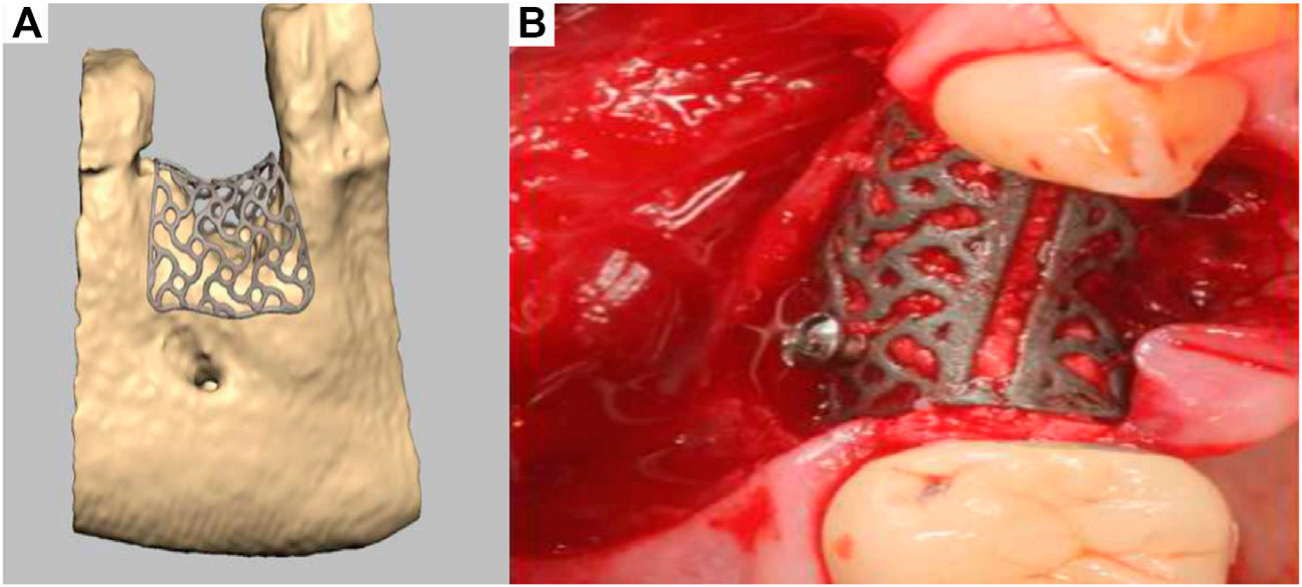
Titanium 3D-printed mesh for vertical bone augmentation. **(A)**: numerical model of the titanium mesh, **(B)**: clinical placement. Reproduced with permission from (Seiler et al., 2018).

A recent development has seen the emergence of a patient-specific 3D-printed titanium mesh for vertical bone augmentation (Seiler et al., 2018). This system, first patented in 2013, utilizes the most recent development of CAD/CAM and metal additive manufacturing technology. This product is designed using CT or CBCT patient data and numerically processed in order to fabricate a mesh that is patient specific and therefore follows the natural contours of the patient using a workflow similar to that previously described (Bartnikowski et al., 2020). Developed by ReOss and distributed by Geistlich, this product called Yxoss CBR enables clinicians to implant patient specific, anatomically accurate titanium cages. The cage is filled with bone grafting materials such as anorganic bone graft and/or autologous bone particles and provides the necessary space maintenance for extraskeletal bone formation to occur. Interestingly, the medical device can significantly reduce the length of the surgery and can be readily biomechanically fixed using conventional titanium screws. As shown in Figure 11A, a numerically created anatomical model of the scaffold is generated for 3D-printed manufacturing, then implanted in conjunction with bone grafting materials (Figure 11B). Upon surgical re-entry the titanium cage is removed in order to enable dental implant placement. This technology is already in clinical use, and while it represents a breakthrough for vertical bone regeneration, it is relatively recent and ongoing clinical studies to verify and quantify the efficacy of this approach for vertical bone augmentation are required. Indeed, recent case reports have shown that this approach can yield favourable clinical outcomes (Dellavia et al., 2021), although complications such as transmucosal exposure of the device (Chiapasco et al., 2021) and inaccuracies between planned and created volume and bone height (Li et al., 2021) are common. The exposure of the device is strongly related to the management of the soft tissue healing component of the vertical bone augmentation procedure. In order to mitigate this issue, the utilisation of autologous membrane fabricated from blood may provide a significant advantage as recently reported (Hartmann and Seiler, 2020). Advanced Plasma Rich Fibrin (A-PRF) membrane is obtained from low *g* centrifugation of blood and proven to increase soft tissue healing (Miron et al., 2017; Miron et al., 2020). As a result, the utilisation of an A-PRF membrane over the titanium patient-specific mesh significantly reduced the frequency of exposure. Therefore, the utilisation of soft tissue healing membranes in conjunction with the Yxoss system may result in better soft tissue outcome but require further investigation. In addition to the exposure of the medical device, a clear limitation of the use of these titanium meshes is the requirement for a second surgical procedure to remove the device. This could be circumvented by the utilisation of biodegradable polymers or degradable metals (Venezuela et al., 2019; Carluccio et al., 2020).

## Conclusion

This review has described the major 3D-printing strategies for achieving extraskeletal bone formation: 3D-powder printing of bio-ceramic, bio-ceramic extrusion 3D-printing, fused deposition modelling using polymer or a mixture of polymer and inorganic filler, and metal 3D-printing. While each strategy is respectively limited by brittleness, the lack of bioactivity, and the requirement of removing non-degradable devices, it is clear that the future of the field lies with the manufacturing of patient specific geometries. The ideal anatomically accurate construct will promote extraskeletal bone formation and provide long-term space maintenance in order to allow for multiple cycles of bone remodelling, towards preventing bone resorption upon implant placement, thus ensuring implant longevity.
